# Incidence of severe subcutaneous emphysema after robotic versus conventional laparoscopic surgery: A retrospective single-center study

**DOI:** 10.20407/fmj.2025-004

**Published:** 2025-08-06

**Authors:** Satoshi Komatsu, Tomoyuki Nakamura, Naohide Kuriyama, Takahiro Kawaji, Osamu Nishida

**Affiliations:** Department of Anesthesiology and Critical Care Medicine, Fujita Health University, School of Medicine, Toyoake, Aichi, Japan

**Keywords:** Laparoscopic surgery, Subcutaneous emphysema, Robotic surgery, Complications

## Abstract

**Objectives:**

Subcutaneous emphysema (SE) in pneumoperitoneum surgery is a complication not observed in open abdominal surgery, but data are scarce and epidemiology is inadequate. Although SE is considered less problematic because it is caused by carbon dioxide gas, past reports have shown that severe complications can occur, such as airway obstruction and cardiopulmonary collapse. We conducted an exploratory single-center retrospective study of patients admitted to the ICU after robotic or conventional laparoscopic abdominal surgery, focusing on epidemiological factors promoting severe SE.

**Methods:**

This retrospective study examined cases of severe SE following robotic or conventional laparoscopic abdominal surgery that necessitated ICU admission. Patients older than 18 years with unscheduled ICU admission for grade 5 head SE between 1 January 2010 and 31 December 2021 were included. All robotic surgeries used the da Vinci Surgical System.

**Results:**

We reviewed data from all 8 of 3,532 robotic surgeries and 5 of 10,305 conventional laparoscopic surgeries resulting in severe SE. By approach, the incidence of severe SE was 0.23% for robotic surgery and 0.05% for laparoscopic surgery (p=0.006). By organ, gastric surgery was the leading cause, with 6 cases from 2,224 gastric surgeries and 7 from a total of 11,613 other abdominal surgeries, giving incidences of 0.27% and 0.06%, respectively (p=0.011). The incidence in women was 0.15% (10/6,496), compared with only 0.04% (3/7,341) in men (p=0.048).

**Conclusions:**

Our findings confirmed that higher risk of severe SE—although still small—is associated with robotic and gastric surgeries and female sex.

## Introduction

Laparoscopic surgery has become a widely used alternative to open surgery. Robotic laparoscopic surgery (referred to as “robotic surgery” hereafter) can, in certain complex cases, replace conventional laparoscopic surgery (“laparoscopic surgery” hereafter), achieving superior outcomes in some areas.^1,2^ However, comparisons of pneumoperitoneum-related complications between these approaches remain limited.

Subcutaneous emphysema (SE), which occurs when gas enters the subcutaneous and soft tissues,^3^ has been observed in 2.3% of laparoscopic surgeries.^4^ SE is typically benign but can lead to serious complications such as hypercarbia and the resulting acidosis.^5,6^ Although rare, SE can also spread extensively beyond the neck, reaching the hypopharynx and potentially causing fatal upper airway obstruction.^7–9^ To investigate severe cases of SE extending to the head—classified as grade 5^3^—we conducted an exploratory, single-center retrospective study of patients admitted to the intensive care unit (ICU) following laparoscopic or robotic abdominal surgery, focusing on epidemiological factors that may promote severe SE.

## Methods

### Ethical approval

This study was approved by the Ethics Committee of Fujita Health University (approval number: HM22-258). Informed consent was waived because of the retrospective nature of the study.

### Patients’ criteria

The study participants comprised all consecutive patients admitted to the ICU between 1 January 2010 and 31 December 2021 for grade 5 SE, from a total of 3,532 robotic and 10,305 laparoscopic abdominal surgeries. SE was assessed immediately after surgery using X-ray imaging, and all patients diagnosed with grade 5 SE were admitted to the ICU. The region and severity of SE were confirmed postoperatively by X-ray and palpation. Patients under 18 years of age and those who underwent esophageal or respiratory surgery were excluded. All robotic procedures were performed using the da Vinci Surgical System (Intuitive Surgical, Sunnyvale, CA, USA).

### Data collection

Data were retrospectively extracted from the patients’ electronic medical records, including ICU records. Collected variables included age, sex, height, weight, body mass index (BMI), American Society of Anesthesiologists physical status, steroid use, collagen disease, emphysema, prior open abdominal surgery, and anesthesia-related factors such as insufflation time, intraoperative fluid balance, surgical site, number of operative ports, use of robotic surgery, air volume (including estimated), performance of extubation in the operating room, and other relevant factors. For patients who were not extubated in the operating room, additional data included the duration of ventilator management from the end of insufflation to extubation, blood gas values at ICU admission, and minute volume (MV). The ratio of measured to estimated minute ventilation was defined as 1 when the patient was ventilated at a tidal volume of 8 mL/kg (based on ideal body weight) with a respiratory rate of 16 breaths per minute, which was used as the estimated MV. Decisions regarding extubation in the operating room for patients with SE were made by the anesthesiologist, while post-ICU admission extubation decisions were managed by intensivists. Continuous variables are presented as median [interquartile range].

### Statistical analysis

Statistical assessments were conducted using Fisher’s exact test for nominal variables and the Mann–Whitney U test for continuous variables, with a significance threshold of p<0.05. All analyses were performed using EZR (Saitama Medical Center, Jichi Medical University, Japan), a graphical user interface for R (The R Foundation for Statistical Computing, Vienna, Austria).^10^

## Results

In total, 13,837 surgeries (3,532 robotic and 10,305 laparoscopic) were included, involving 7,371 men and 6,496 women. Severe SE developed in 3 men (0.04%) and 10 women (0.15%) (p=0.048) (Table 1). The incidence of SE was 0.23% (8/3,532) for robotic surgery and 0.05% (5/10,305) for laparoscopic surgery, representing a 4.6-fold higher rate for robotic procedures (p=0.006) (Table 1). Thirteen cases of severe SE were analyzed (Table 2), excluding two cases in which the extent of SE was unknown. Most patients were small, thin women of advanced age (median age, 77 [75, 80] years; height, 149 [148, 162] cm; BMI, 18.1 [17.7, 21.0] kg/m^2^; 77% female) (Table 2). None had a history of steroid use or chronic lung disease, and only one had collagen disease. The median surgical time was 567 [428, 605] minutes, and the median insufflation time was 537 [366, 559] minutes. Gastric surgery was the most common procedure, accounting for 6 of the 13 patients (46%) with severe SE, followed by uterine and rectal surgery in 2 patients each and colon, liver, and pancreatic surgery in 1 patient each. The non-gastric procedures are collectively referred to as “other surgeries.” Severe SE was significantly more common after gastric surgery (6/2,224=0.27%) than after other surgeries (7/11,613=0.06%) (p=0.011) (Table 1). The median number of operative ports was 5 [5, 5] (range, 4–6), and the median gas consumption (including estimates) was 899 [718, 1132] L. No significant differences in patient background factors were observed when comparing gastric versus other surgeries (Table 3A) or robotic versus laparoscopic surgeries (Table 3B).

Three patients were extubated in the operating room. Airway evaluations performed intraoperatively included direct laryngoscopic exposure in 2 cases, cuff leak test confirmation in 2 cases, blood gas analysis in 11 cases, and clinical judgment by the anesthesiologist in 1 case. Among the 10 patients not extubated in the operating room, arterial blood gas values at ICU admission showed a pH of 7.45 [7.41, 7.46], PaCO_2_ of 34.4 [33.4, 39.2] mmHg, measured MV of 6.4 [6.0, 7.5] L/min, and estimated MV of 6.6 [6.0, 7.4] L/min. The time from the end of insufflation to the start of ventilator management was 979 [873, 1030] minutes. All patients were extubated within 1 day after ICU admission, and ICU stays ranged from 2 to 3 days. None of the patients required reintubation following extubation.

## Discussion

Our single-center, exploratory, retrospective study of patients with unscheduled ICU admissions for severe SE following robotic or laparoscopic surgery aligns with previously observed trends in risk factors. For instance, a higher incidence of SE, including grade 5 SE, has been reported after robotic surgery,^11^ and particularly high incidences have been noted following gastric surgery.^12^

The overall occurrence of SE in robotic and laparoscopic procedures has generally been documented at below 3%.^4,11,13,14^ However, rates of cervical SE have been reported as 3.62% in robot-assisted rectal surgery,^11^ 5.24% in robot-assisted total prostatectomy,^13^ and 5.9% in robot-assisted general, urological, and gynecological surgeries.^15^ At the high end, robotic gastric surgery has shown a wide range, with cervical SE rates reported as high as 21%.^12^ These incidences are considerably higher than the percentages found in the present study, and the variability may be attributable to differences in surgical techniques.

In our results, gastric surgery was the most common cause of grade 5 SE, accounting for nearly half of the cases. Despite the long operative times associated with gastric procedures, no significant differences in patient background or operative factors were observed compared with non-gastric surgeries. The reverse Trendelenburg position used during gastric surgery may contribute to the upward spread of SE to the neck, although this remains speculative. At present, risk stratification based on surgery type or underlying disease has not been established, and a previous report found no significant association between the type of surgery and the occurrence of SE.^14^ Severe SE remains rare in obstetrics and gynecology.^16^

Different robotic surgery systems have distinct characteristics. In this study, all robotic procedures were performed using the da Vinci system, which includes specific features related to pneumoperitoneum management, such as its docking port and abdominal wall lifting mechanism. These features may have contributed to the incidence of severe SE.

Recent reports have identified several SE risk factors, including age of >65 years, female sex, lean BMI, high gas volume, use of more than six operative ports, operative time of ≥200 minutes, pneumoperitoneal pressure of ≥15 cmH_2_O, steroid use, and chronic lung disease.^13,14^ Our findings are consistent with these because most patients were thin women over the age of 65 years. All 13 surgeries in our series exceeded 200 minutes in duration, although only 2 involved 6-port surgery. Few patients had fragile tissues, such as those with steroid use or collagen disease, and preoperative respiratory tests were normal.

Serious adverse events from minor SE and hypercarbia are rare but can lead to surgical interruption, conversion to open surgery, or reintubation.^4,7,8,15^ Reintubation after extubation following general anesthesia has been reported in only 0.03% of cases,^17^ whereas one study found a 1.7% rate of failed extubation due to cervical SE.^15^ In the present study, no patients required reintubation after extubation. Ten patients (77%) were admitted to the ICU while still under ventilatory management, suggesting that maintaining intubation until the SE subsided may have been effective. Blood gas analyses collected at ICU admission from patients who remained intubated showed no signs of acidosis or hypercarbia. This suggests that despite the presence of grade 5 SE, extubation might have been physiologically feasible as indicated by the blood gas results. The decision to delay extubation may have been driven more by clinical caution than by physiological necessity. Further research is needed to establish clear criteria for extubation in such cases.

### Limitation

This was a single-center, retrospective study. It included only patients who were admitted to the ICU unscheduled for management of severe SE, and there were no standardized criteria for ICU admission or extubation. Because these decisions were left to the discretion of individual anesthesiologists, a uniform approach cannot be assumed. We did not evaluate the complete clinical backgrounds of the patients, and other unidentified factors may have contributed to the risk of developing severe SE. Additionally, we were unable to verify certain intraoperative details, such as temperature, ventilator settings, and end-tidal carbon dioxide levels during anesthesia.

## Conclusions

Severe SE induced by pneumoperitoneum surgery was found to occur more frequently in robotic surgery, in gastric procedures, and in women. These findings are consistent with the limited data currently available on this topic, despite some quantitative differences across studies. Future research should aim to clarify the key factors driving these trends and investigate whether treatment protocols contribute to differences between hospitals and surgical approaches—and whether these protocols can be optimized.

## Figures and Tables

**Table 1:** Characteristics of all patients

	Severe SE (N=13)	Non-severe SE (N=13,827)	p value
Surgery (robotic:laparoscopic), n	8:5	3,524:10,300	0.006
Age, years	77 [75, 80]	63 [47, 72]	0.002
Sex (male:female), n	3:10	7,338:6,486	0.048
Type of organ (gastric:other), n	6:7	2,218:11,606	0.011
Operation time, min	567 [428, 605]	188 [129, 284]	<0.001
Numbers of robotic and laparoscopic surgeries by organ			
	Robotic surgery (N=3,532)	Laparoscopic surgery (N=10,305)	p value
Gastric surgery, n (severe SE, n)	714 (5)	1,510 (1)	0.002
Other abdominal surgeries, n (severe SE, n)	597 (2)	5,442 (3)	0.08
Gynecological surgery, n (severe SE, n)	262 (1)	2,698 (1)	0.17
Urological surgery, n (severe SE, n)	1,959 (0)	655 (0)	1

Data are presented as number of patients or median [interquartile range]. The table shows comparisons between patients with severe SE and other patients (upper part of the table) and between the numbers of robotic and laparoscopic surgeries by organ (lower part of the table). SE, subcutaneous emphysema

**Table 2:** Characteristics of patients with severe SE

	Patients with severe SE (N=13)
Age, years	77 [75, 80]
Sex, male:female	3:10
Height, cm	149 [148, 162]
Weight, kg	48 [39, 53]
BMI, kg/m^2^	18.1 [17.7, 21.0]
ASA physical status	2 [1, 2]
Steroid therapy	0 (0)
Collagen disease	1 (8)
Chronic lung disease	0 (0)
Laparotomy history	3 (23)
Diabetes	2 (15)
Hypertension	5 (38)
Robotic surgery	8 (62)
Operative ports, n	5 [5, 5]
Insufflation time, min	537 [366, 559]
Total CO_2_ gas, L^a^	899 [718, 1,132]

Note: Some listed data are also shown in Table 1, but are shown here again for convenience. Data are presented as median [interquartile range] or n (%). ^a^ Estimated from insufflation time and set pressure if not provided. SE, subcutaneous emphysema; BMI, body mass index; ASA, American Society of Anesthesiologists

**Table 3:** Characteristics of patients with severe SE cases by surgery type: (A) Gastric vs. other and (B) Robotic vs. laparoscopic

(A) Characteristics of patients with severe SE undergoing gastric or other surgery			
Characteristics	Gastric surgery (N=6)	Other surgery^a^ (N=7)	p value
Age, years	78 [76, 80]	76 [48, 81]	0.615
Sex, male:female	2:4	1:6	0.559
Height, cm	149 [147, 158]	153 [148, 164]	0.388
Weight, kg	41 [38, 47]	49 [43, 54]	0.295
BMI, kg/m^2^	18.1 [17.5, 19.4]	20.2 [17.8, 22.0]	0.431
ASA physical status	2 [2, 2]	1 [1, 2]	0.177
Collagen disease	1 (17)	0 (0)	0.462
Laparotomy history	3 (50)	0 (0)	0.0699
Hypertension	4 (67)	1 (14)	0.103
Robotic surgery	5 (83)	3 (43)	0.266
Operative ports, n	5 [5, 5]	5 [4, 5]	0.218
Insufflation time, min	543 [407, 615]	537 [210, 546]	0.445
Total CO_2_ gas, L^b^	745 [711, 1,024]	1,105 [822, 1,155]	0.534
(B) Characteristics of patients with severe SE undergoing robotic or laparoscopic surgery			
Characteristics	Robotic surgery (N=8)	Laparoscopic surgery (N=5)	p value
Age, years	78 [70, 80]	76 [75, 79]	0.659
Sex, male:female	2:6	1:4	1
Height, cm	149 [148, 161]	153 [148, 163]	0.555
Weight, kg	41 [38, 50]	48 [48, 55]	0.354
BMI, kg/m^2^	18.1 [17.5, 19.4]	20.2 [17.8, 22.0]	0.431
ASA physical status	2 [1, 2]	1 [1, 2]	0.415
Collagen disease	1 (13)	0 (0)	1
Laparotomy history	2 (25)	1 (20)	1
Hypertension	5 (63)	0 (0)	0.075
Gastric surgery	5 (63)	1 (20)	0.266
Operative ports, n	5 [5, 5]	5 [5, 5]	0.801
Operation time, min	571 [526, 618]	438 [244, 605]	0.724
Insufflation time, min	541 [486, 578]	367 [216, 547]	0.622
Total CO_2_ gas, L^b^	939 [736, 1125]	899 [718, 1132]	0.943

Data are presented as n, n (%), or median [interquartile range]. ^a^ Other surgeries included uterine (n=2), rectal (n=2), colon (n=1), liver (n=1), and pancreatic (n=1) surgeries ^b^ Estimated from insufflation time and set pressure if not provided. SE, subcutaneous emphysema; BMI, body mass index; ASA, American Society of Anesthesiologists
